# Conservative Management of Segmental Infarction of the Greater Omentum: A Case Report and Review of Literature

**DOI:** 10.1155/2010/765389

**Published:** 2010-09-19

**Authors:** Ramawad Soobrah, Mohammad Badran, Simon G. Smith

**Affiliations:** ^1^Undergraduate Department, Northwick Park Hospital, Harrow HA1 3UJ, UK; ^2^Department of Radiology, Broomfield Hospital, Essex, UK; ^3^Department of General Surgery, Broomfield Hospital, Essex CM1 7ET, UK

## Abstract

Segmental omental infarction (SOI) is a rare cause of acute abdominal pain. Depending on the site of infarction, it mimics conditions like appendicitis, cholecystitis, and diverticulitis. Before the widespread use of Computed Tomography (CT), the diagnosis was usually made intraoperatively. SOI produces characteristic radiological appearances on CT scan; hence, correct diagnosis using this form of imaging may prevent unnecessary surgery. We present the case of a young woman who was treated conservatively after accurate radiological diagnosis.

## 1. Introduction

Segmental infarction of the greater omentum was described over 100 years ago [1]; however, the aetiology is still unknown [2, 3]. Most patients present with right-sided abdominal pain (90%), and males are more frequently affected (ratio 2 : 1) [3, 4]. It has been postulated that the right side of the omentum is more susceptible to infarction due to its greater length and mobility [5]. Other authors have attributed its occurrence to a different embryonic origin of the right side of the omentum with congenitally anomalous fragile blood vessels [6, 7]. This condition occurs mainly in people in their fourth and fifth decades [8], and a significant proportion of cases have also been described in the paediatric population (15%) [9].

## 2. Case Presentation

**Figure 1:**
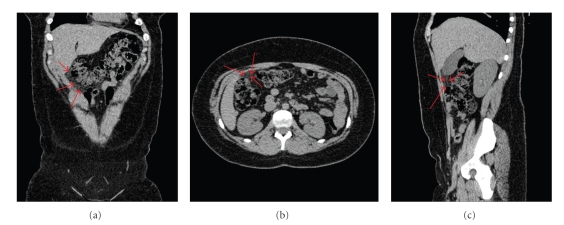
Unenhanced CT images (a) coronal, (b) axial, (c) sagittal show a focal area of hyperattenuating omental fat stranding (arrows).

A 20-year-old woman without significant previous medical history presented with a one-week history of acute right-upper quadrant (RUQ) pain and no other gastrointestinal symptoms. Examination revealed focal tenderness in the RUQ with mild peritonism. Murphy's sign was negative. She was apyrexial; pregnancy test was negative; routine blood investigations revealed a raised white cell count of 13.6 × 10^3^/ml and a C-reactive protein (CRP) of 88 mg/dl. Other blood tests and erect chest radiograph were unremarkable. Microscopic haematuria was also noted on urinalysis. An unenhanced CT scan was subsequently performed and demonstrated a focal region of heterogenous increased fat density involving the right omentum between the hepatic flexure and anterior abdominal wall (Figure 1). No other abnormalities were found, and based on these CT findings, a diagnosis of SOI was made. The patient was closely observed and managed conservatively with analgesia. Her abdominal pain gradually resolved, and she was discharged three days after hospitalisation.

## 3. Discussion

The incidence of SOI is estimated to be around 0.1% of all laparotomies performed for acute abdomen [4]. Various predisposing factors have been implicated including obesity, trauma, recent abdominal surgery, postprandial vascular congestion, sudden increase in intraabdominal pressure, and hypercoagulability [8, 10–12]. Table 1 shows the classification of segmental infarction of the greater omentum.

Clinical findings for SOI tend to be nonspecific. Patients are constitutionally well and present with acute or subacute abdominal pain; gastrointestinal symptoms such as nausea, vomiting, anorexia, and diarrhoea are uncommon [5, 12]. Temperature is usually normal or slightly raised; there is localised tenderness with varying degree of guarding on the right side of the abdomen [13]. The white blood cell count and CRP may be elevated [8]. Therefore, omental infarction is difficult to be distinguished clinically from common surgical ailments such as appendicitis and cholecystitis. 

Correct radiological diagnosis is important to establish the most appropriate treatment plan for the patient. Ultrasound scan (USS) or computed tomography can be used to make a reliable diagnosis. Typical CT findings of omental infarction include a well-circumscribed ovoid area of heterogenous fat stranding with hyperattenuating streaks located within the omentum between the rectus abdominis and colon [8, 11]. Typical features found on ultrasound scan include a moderately hyperechoic noncompressible lesion at the site of maximal tenderness [5]. In the past, diagnosis of SOI was rarely made preoperatively. The routine use of CT and USS in the assessment of acute abdominal pain coupled with improved awareness of this condition may account for the increasing number of cases being identified [11, 14]. 

Omental infarction can either be managed conservatively or surgically, and there are controversies about the correct treatment modality. Some authors recommend surgical intervention because it leads to a shorter hospitalisation period and a more rapid improvement of the patients' pain [15–19]. Also, there is less risk of rare complications including abscess formation, adhesions, and sepsis [11, 20, 21]. 

Others argue that unnecessary operations should be avoided because this disease usually runs a self-limited course [3, 18, 21–23]. Using PubMed, a review of the English literature regarding conservative management of SOI was performed for the period from 1990 to 2010. Twenty-one relevant articles with a total of 64 patients were identified. All patients underwent USS and/or CT to confirm the diagnosis of SOI. The patient details for these studies are summarised in Table 2. 

Fifty-four patients underwent successful conservative management with no ensuing complications. However, 10 patients (15.6%) had subsequent laparoscopic resection of the infarcted omentum having failed conservative management. The reasons for failed initial conservative treatment were severe intractable pain and worsening of symptoms. The postoperative recovery of these patients was uneventful. The results from the above table demonstrate that non-operative treatment of omental infarction has been achieved in several series with successful outcomes provided that an accurate radiological diagnosis is obtained and the patient's condition remains stable. Moreover, with conservative management, most patients become symptom-free within two weeks [2, 5, 31]. 

Surgical exploration of the abdomen is mandatory in patients with unclear radiological findings or if the patient's clinical condition deteriorates [3]. If surgical intervention is required, then laparoscopic exploration should be considered as it can be both diagnostic and therapeutic and are associated with low morbidity [10, 20, 37, 38]. Of note, up to half of cases of omental infarction is associated with free serosanguineous peritoneal fluid [7, 9, 13]. The presence of this fluid and normal intraabdominal viscera should encourage further exploration and closer inspection of the omentum during laparoscopy [39].

## 4. Conclusion

Segmental omental infarction is a benign rare cause of acute abdomen, and hence it is seldom considered in the differential diagnosis of acute abdominal pain. It mimicks symptoms that often leads to misdiagnosis of appendicitis, cholecystitis, or diverticulitis [14]. The use of cross-sectional imaging allows us to obtain typical, well-recognisable, and reliable imaging features to diagnose this entity and hence avoid unnecessary surgery. Moreover, complications of conservative management tend to be rare. The main disadvantages of conservative management are longer hospitalization and the increased use of analgesics [22]. The standard treatment modality for omental infarction has not been fully established to date; however, most cases diagnosed radiologically have been followedup by surgical intervention [2]. We believe a conservative treatment approach is justified in the majority of patients affected by SOI after thorough imaging evaluation and the exclusion of other significant intraabdominal pathologies.

## Figures and Tables

**Table 1:** Classification of omental infarction [2, 8, 13].

Torsion-related	Nontorsion-related (thrombosis)
– Primary (idiopathic)	– Spontaneous infarction
– Secondary to adhesions, hernias, or tumours	– Hypercoagulable states
	– Vascular abnormality
	– Trauma

**Table 2:** Summary of patient demographics.

Successful conservative management (**n** = 54)	Failed conservative management (*n* = 10)
[3, 11, 18, 21–35]	[3, 8, 18, 19, 22, 23, 36]
Adults = 23	Adults = 7
Children = 7	Children = 3
Unknown = 24	

Male = 25	Male = 7
Female = 12	Unknown = 3
Unknown = 17	

Male average age = (739/19) = 39 years	Average age = (254/7) = 36 years
Female average age = (354/11) = 32 years	
